# “This Is Not the Original Timeline”: A Case Report of an Extended Dissociative Episode in a Healthy Young Male Accompanied with Severe Decline in Mental State

**DOI:** 10.1155/2021/6619579

**Published:** 2021-05-03

**Authors:** Godwin Tong, Kieran Groom, Louisa Ward, Muhammad Naeem

**Affiliations:** Holt Ward, The Elgar Unit, Newtown Hospital, Worcestershire Health and Care Trust, Newtown Road, Worcester, Worcestershire WR5 1JG, UK

## Abstract

Dissociation is a disconnection between a person's thoughts, memories, feelings, actions, or sense of who he or she is. Dissociative disorders can be described and understood using the combination of five core symptoms: amnesia, depersonalisation, derealisation, identity confusion, or identity alteration. They are frequently associated with previous experience of trauma. The challenge in diagnosis and the lifetime prevalence of approximately 10% in the general population and clinical psychiatric setting ensures the relevance of this case. We write about a 21-year-old gentleman with history of autism and obsessive compulsive disorder, but no significant medical history was presented to the emergency department with increased anxiety, subsequently progressing to agitation, pacing, and becoming nonverbal. No significant findings were uncovered on laboratory blood testing (other than prolactin 737 mu/L and phosphate 0.35 mmol/L), lumbar puncture, or brain imaging. Consequently, he was admitted to a psychiatric unit for assessment. The patient continued to present with severe disorientation, limited speech, and altered state of consciousness with occasional spastic-like movements. Antipsychotic and benzodiazepine medication was initiated, with no significant change in presentation. The patient continued to be witnessed wandering and having incoherent speech. First signs of improvement came 21 days postadmission with brief conversation and lucidity. This continued to improve over the next 7 days where he was reported to be at his baseline mental state. Environmental stressors including university examinations, the COVID-19 pandemic, and recent contact with his estranged father were possible precipitants to the episode. The patient reported almost complete unawareness of the psychiatric admission. A diagnosis of dissociative disorder, unspecified, was given. This case shows the management and diagnostic challenges of patients presenting with the aforementioned symptoms. There are no formal guidelines for the management of treating dissociative episodes, and this case report suggests the possible benefits of a drug-free period of watchful waiting upon admission.

## 1. Introduction

Dissociative (conversion) disorder (F44, ICD-10) is an umbrella term overarching various subtypes of dissociative disorders like but shares the common themes of partial or complete impairment of memories, awareness of identity, sensations, or control of body movements. From a medical perspective, patients with dissociative disorders have no physical or neurological problems. Episodes are often associated with traumatic life events or psychological stress. Dissociative disorders are underrecognised in general, and dissociative (conversion) disorder, unspecified (F44.9), is even less prevalent; hence, it is no surprise that limited literature exists around the subject. Available studies have shown that dissociative disorders have a lifetime prevalence of about 10% in the general population and clinical psychiatric setting [1]. Dissociative Identity Disorder (DID) (F44.8) is the most pervasive and most common out of the group of dissociative disorders, forming up to 5%, leaving the remaining 5% split between the lesser reported subtypes [2]. The onset of dissociative episodes is usually sudden and usually follows traumatic or stressful events [2]. Duration of dissociative episodes can range from hours to days but typically not more than 2 weeks [3–7]. Here, we present a case of a young male who experienced a dissociative episode over a prolonged period of time—4 weeks, with a unique mix of psychiatric and behavioural symptoms including formal thought disorder accompanied with severe thought block and word salad, purposeless wandering, chaotic behaviour, and bizarre delusions. Whilst we had various initial differential diagnoses, we explain later in the discussion section why the other differentials are unlikely and how this mix of symptoms has prompted us to give him a diagnosis of dissociative (conversion) disorder, unspecified (F44.9).

## 2. Case Report

A 21-year-old male initially presented to the Accident and Emergency (A&E) department of a district general hospital with increased anxiety reporting that they felt like he was dying. He had a background of high functioning autism, obsessive-compulsive disorder (OCD), and no significant physical health problems. In terms of family history, he was the only child and had no significant family history of medical nor psychiatric illnesses. He admitted to using cocaine that day but subsequent collateral history from his aunt, whom he was staying with at the time denied this. A&E doctors diagnosed him as having a panic attack and discharged him shortly after back to his aunt's farm. Within hours, he presented to A&E again with increased signs of agitation, pacing around, and being nonverbal. As such, no further history could be obtained from him. Initial bloodwork from A&E looking at full blood count, liver function, renal profile, and inflammatory markers were normal. Urine toxic substance screen was negative. The only positive findings were low phosphate 0.35 mmol/L and raised prolactin 737 mu/L despite no previous antipsychotic use. X-ray of the skull revealed no fractures. At this time, differential diagnoses included meningitis, encephalitis, psychosis, and delirium. MRI head subsequently revealed a normal-sized pituitary gland, and no specific abnormalities were seen intracranially. A&E then started treatment for IV acyclovir for prophylaxis against potential encephalitis. He was moved to the acute medical unit where his hypophosphataemia was treated with oral phosphate replacement and levels returned to normal shortly after. A week later, lumbar puncture analysis returned negative for bacteria and viruses. Due to this unusual presentation, he was referred to the mental health liaison of the acute psychiatric unit for mental health assessment and was formally admitted to our acute psychiatric unit for treatment 9 days after initial presentation.

By the time of admission, he was displaying severe disorientation to time, place, and person. Thought block was evident and major, accompanied by markedly limited speech. He appeared to be in an altered state of consciousness and had a dazed confused look, accompanied with an almost catatonic-like physical presentation whereby there was increased muscle tone in upper and lower limbs bilaterally but was able to withdraw sharply to needles when doctors attempted phlebotomy. Movements were few and spastic. There were no features associated with an ictal episode. To quote some of his word salad—“this is not the original,” “this is not the right timeline,” “two people fighting,” “two people joined in the middle, different timelines.” He did on occasion seem able to answer a yes/no question but with great effort and could never remember what he said. He was still able to withdraw from the pain from an attempted venepuncture. Mental state examination demonstrated a relatively well-kempt young man who had poor eye contact but when eye contact was made, displayed an intense stare. His speech was of normal tone and volume but very stilted. Contents of speech were disjointed and bizarre. Mood was subjectively and objectively euthymic. Thoughts were considered to be very disordered and chaotic. From what we could make out of his jumbled and limited speech, it seemed like he was occasionally describing two people arguing, possibly suggesting auditory hallucinations. He was disorientated to time, person, and place, and he lacked insight and capacity with regard to his mental state.

We then prescribed him lorazepam 1 mg four times daily and quetiapine 50 mg daily with a plan of increasing it to 300 mg in five days in the hope of ruling out psychosis. Five days later, there was no change in presentation in this patient as he continued to display symptoms of thought block and jumbled speech. From what we could discern from his speech, it seemed like he had delusions about him not living in the correct timeline; his DNA is not being his own, and his mother was not his original birth mother. He did however start to mobilise and walked around the ward albeit with a slightly spastic gait. Behaviour remained bizarre as he was often found removing his clothes and walking around aimlessly on the ward. As quetiapine did not appear to be working, we decided to replace quetiapine with haloperidol 5 mg once daily and to replace lorazepam with diazepam 10 mg three times daily. We then added sertraline 50 mg once every morning. Unfortunately, he shortly developed some extrapyramidal side effects (EPSEs)—leg stiffness from haloperidol, and we replaced it with aripiprazole 10 mg once daily after three days. Procyclidine 5 mg three times daily was found to be helpful in treating the EPSEs.

One week later, we repeated blood tests which revealed a continued rise of prolactin and reduced parathyroid hormone as the only positive findings. Upon discussion with the endocrinology department, the rising trend of prolactin was believed to be secondary to antipsychotic use. Furthermore, since the recent MRI was unremarkable, we felt it did not warrant further investigation. Blood was also negative for NMDA receptor antibodies, Treponema pallidum antibodies, hepatitis B and C antibodies, and HIV 1&2 antigen/antibodies. By the 19^th^ day postadmission, presentation remained unchanged except with the addition of increased frequency of agitation episodes which include but not limited to using excessive strength to hold onto staff, throwing of ward furniture, not complying with staff instructions, and trying to access staff areas with force. In view of this, we increased aripiprazole dose to 20 mg once a day. He continued to be wandering aimlessly on the ward, speech remained mainly incoherent, but word range had expanded slightly to being able to speak half sentences. For example, “Jesus, these are genuinely not one of the original sounds or thoughts or sound tracks,” “These are both our original thoughts. I think it was all of them, there was no, this is wait, there was no original timeline,” “I am not the soft science in the Jurassic thoughts.” Despite no improvement in this thought block after prescribing aripiprazole, we did notice a reduction in agitation episode frequency and severity; hence, we decided to persist with the medication.

First signs of improvement only came about 21 days after admission where nurses observed a moment of lucidity where the patient was able to speak full sentences and engage in a brief conversation but returned to his confused state shortly after. On the 22^nd^ day, the patient suddenly appeared calm and regained some cognitive orientation to person and place. He was able to hold conversations, but as the day progressed, his ability to retain information and overall mental state declined and was observed to be asking the same questions repetitively such as when he was going to be seen by doctors. On the 23^rd^ day, the patient did not display any of the previous symptoms and his mental state appeared to almost match that of baseline with the exception of reduced concentration levels. He was able to engage with staff for most of the day and was even observed to be using his personal laptop computer at one point in time. On the 28^th^ day, the patient was reviewed and it was decided that the patient was not displaying any acute signs of mental illness and was able to perform all activities of daily living independently. It was thus decided that he was fit for discharge. In conversation, the clinicians asked him if he had any awareness of the past few weeks and he displayed localised episodic amnesia by not being able to recall anything in the past few weeks. COVID-19 antibodies and nasal swab results were negative.

## 3. Discussion

Here, we present a case of a young male who suffered from a dissociative episode for an extended period of about 28 days. The case piqued our interest in its duration and severity of the associated symptoms. The patient suffered from severe functional impairment and decline of mental state as seen from the purposeless wandering, thought block, and delusional word salad, yet still retaining the ability to perform certain activities of daily living like eating his meals independently. It is also important to consider several aspects of this patient's background and social history and discuss the thoughts leading to this diagnosis. He was born in England but moved to Gibraltar early on and spent his formative years there. His parents separated when he was nine years old, and he has a family history of Asperger's syndrome. Socially, the patient is highly intelligent from birth; it was reported by his mother that he had a reading age of four at 18 months and he recently graduated from a top British university. However, he did have an extensive recreational drug history involving cocaine, amphetamines, cannabis, and Ritalin. Our differential diagnoses included the following: encephalitis, meningitis, drug-induced psychosis, delirium, dissociative amnesia (F44.0), and dissociative (conversion) disorder, unspecified (F44.9).

Both meningitis and encephalitis were easily ruled out with lumbar puncture showing normal protein and glucose levels. Autoimmune encephalitis was also ruled out with a negative array of antibodies against GABA/AMPA/NMDA/CASPR. Whilst it might be tempting to suggest his presentation to be simply drug induced, urine drug screen on the first day of admission to A&E was positive for benzodiazepines but negative for THC, amphetamines, cocaine, antidepressants, and antipsychotics. The presence of benzodiazepines in his urine was not surprising as he was given a stat dose of lorazepam at A&E when he first presented. We note that the initial hypophosphataemia could lead to electrolyte imbalance-induced delirium and indeed delirium was one of the initial differentials in A&E, but within two days, his phosphate levels were replaced orally and remained normal for the entirety of admission, along with his other bone profile parameters. As such, delirium secondary to hypophosphataemia would be an unlikely diagnosis considering his presentation persisted despite electrolyte levels being at normal levels. With regard to his raised prolactin on presentation, one could systematically rule out each potential cause, i.e., physiological, pharmacological, and pathological. A normal MRI scan showing no gross abnormalities would rule out any pathological cause. From a pharmacological perspective, considering this patient was previously antipsychotic naïve, the hyperprolactinaemia could be explained by his historical recreational opiate drug use, coupled with evidence of acute benzodiazepine use as seen from his initial urine drug screen. It has been reported that both opiate and benzodiazepine use can lead to hyperprolactinaemia via dopaminergic receptor inhibition and GABAergic modulation, respectively [8, 9]. From a physiological perspective, stress would be a potential factor for raising prolactin. Collateral history from his mother reported that the patient was under a lot of stress from preparing for examinations, his usual routine being disrupted due to the nationwide lockdown due to COVID-19, and his estranged father recently coming into contact with him. As such, his hyperprolactinaemia could be due to a mixture of physiological and pharmacological causes. Even so, there have not been any reports that hyperprolactinaemia can directly manifest as delirium thus further convincing us that delirium was not a likely differential. Towards the end of his admission, we reviewed his blood biochemistry again and all parameters returned to baseline with the exception of his prolactin which was 1359 mu/L. However, as he was presenting clinically well, we accepted this as a result of the various antipsychotic medication during this admission.

We interviewed the patient after his dissociative episode, and he described his experience like a never-ending nightmare. Memory loss was episodic as he did not remember majority of the past few weeks and his last memory was entering the A&E department. He was completely unaware of what happened on the acute psychiatry unit ward. Whilst we did not attempt any formal memory assessments, his gross long-term memory appeared intact. When we reminded him some of the phrases he used to say, for example, phrases involving how we belonged to a different timeline or how he seemingly described two people fighting, he was puzzled and did not understand why he would say those things as he was not a fan of science fiction. Dissociative identity disorder was briefly considered due to a certain phrase regarding two people fighting which the patient initially mentioned on his first day of admission. However, we excluded this differential quickly as the patient did not verbalise this statement on subsequent observations. Furthermore, there were no distinct personalities displayed by the patient nor any differing patterns in the way the patient experienced his body or the environment. His symptoms mainly revolve around the blocking of thoughts and impairment of general functioning which we feel did not fulfil the criteria for diagnosing dissociative identity disorder. One might also suggest dissociative amnesia to be a possible differential as whilst the patient did experience memory loss of about four weeks, we felt our patient's symptoms were not best described by the ICD10's criteria of memory loss being the main feature. We feel his unique blend of symptoms of being severely thought blocked as though in a trance-like state, purposeless roaming around the ward, postevent amnesia, feelings of depersonalisation, and alterations in sense of self would support our diagnosis of dissociative disorder, unspecified.

When exploring possible triggers, the patient later admitted to feeling overwhelmed by academic pressures from his postgraduate course applications and a disruption of normal routine by having to be confined indoors during the nationwide lockdown prior to admission. He also added that the falling out with his partner coupled with an unsolicited contact attempt by his father contributed to his anxiety. He usually relies heavily on his mother who lives abroad for anxiety management, but in this episode, it was believed that the compounding of multiple stressful events proved too much for him to handle. This element of stress preceding this patient's presentation might well be a major trigger to this dissociative episode.

Eventually, the recovery of this patient was as quick as its onset. With regard to his management, antipsychotic medication did not appear to have any positive effect on his mental state. Sedation was beneficial in managing agitated episodes, and the patient was even observed to expand his speech range after. As he no longer displayed signs of mental illness, he was discharged to the community psychiatric team. He was discharged with aripiprazole 20 mg OD and lorazepam 1 mg TDS with the plan of weaning it off. We would have liked to arrange for psychotherapy to be delivered as a form of long-term treatment in the community, but unfortunately, the patient moved to a different city and would be under the care of a different trust.

On reflection, whilst the old adage “common things are common” still rings true in general medical practice, we would like to remind clinicians to consider alternative diagnoses, especially when the presentation does not fully meet the diagnosing criteria. In this case, drug-induced psychosis would be a tempting easy diagnosis based on the patient's history of drug use, but on further analysis, his behaviour did not fulfil the criteria of psychosis, and even after cessation of drug use, his symptoms persisted. Thus, we feel that when querying a diagnosis of dissociative disorder, management should ideally involve an element of watchful waiting whilst ruling out other differentials before trialling antipsychotics. Perhaps, antipsychotics can be trialled if more definitive symptoms of psychosis are evident. Whilst benzodiazepine was helpful in treating symptomatic agitation, antipsychotic had little effect on his presentation. We did discharge the patient on aripiprazole, but this was mainly for its mood stabilising effect and for relapse prevention. We understand that watchful waiting might unfortunately be difficult to implement on most acute psychiatric units where there are pressures on the clinical team to “medicate and evacuate” so new patients can be admitted. Since there are no formal medical guidelines other than symptom management in treating dissociative episodes, we would like to report our experience and encourage the use of a drug-free period of watchful waiting.
